# Use of Statin Therapy to Reduce Cardiovascular Risk in Older Patients

**DOI:** 10.1155/2010/915296

**Published:** 2010-06-08

**Authors:** N. K. Wenger, S. J. Lewis

**Affiliations:** ^1^Grady Memorial Hospital, School of Medicine, Emory University, Atlanta, GA 30303, USA; ^2^Department of Medicine, Northwest Cardiovascular Institute, Portland, OR 97210, USA

## Abstract

*Background*. Cardiovascular disease is the principal cause of mortality in older individuals, and more than 80% of deaths due to coronary heart disease or stroke occur in patients over 65 years of age. Hyperlipidemia is one of the main modifiable risk factors for cardiovascular disease. Current guidelines recommend the use of statins to reduce low-density lipoprotein cholesterol to appropriate targets based on an individual's cardiovascular risk, and clearly state that older age should not be a barrier to treatment. Despite extensive evidence demonstrating clear benefit with statin therapy in older individuals, this population remains chronically undertreated. *Scope*. This paper provides an overview of the current evidence available regarding the efficacy and safety of statin therapy to reduce cardiovascular risk in older patients. We use hypothetical case studies to address some of the questions frequently posed by physicians responsible for the cardiovascular health of older patients. * Conclusions*. Various factors may account for the failure to provide appropriate treatment, including a lack of awareness of clinical benefits and perceived safety issues. However, if current guidelines are followed and older patients treated to appropriate LDL-C goals, the likelihood of cardiovascular events will be reduced in this high-risk population. Employing an evidence-based approach to the management of cardiovascular risk in older patients is likely to yield benefits in terms of overall cardiovascular burden.

## 1. Introduction

The incidence of cardiovascular (CV) events increases sharply with age, and the prevalence of CV disease and concomitant risk factors is significantly higher in older individuals [1]. Cardiovascular disease is the principal cause of death in older patients, in that well over 80% of deaths due to coronary heart disease (CHD) or stroke occur in individuals 65 years of age or older [2]. Prevention of CV disease and recurrent CV events in older individuals remains a major challenge.

Hyperlipidemia is one of the main modifiable risk factors for CV disease, and current guidelines recommend statin therapy to reduce low-density lipoprotein cholesterol (LDL-C) levels to appropriate targets based on the individual's level of CV risk [3–6]. The pharmacokinetic profiles of statins have been shown to differ little in young and older subjects. What variations that may exist are not considered clinically relevant, and dose adjustments based on age are not advocated [7–9]. However, while lipid-lowering therapy is widely underutilized in middle age, there is considerably more clinical inertia associated with the use of statins in older patients. This occurs despite extensive evidence of a reduction in CV risk associated with statin treatment in this patient age group.

Historically, data from trials such as the Scandinavian Simvastatin Survival Study (4S) [10], the Cholesterol and Recurrent Events (CARE) trial [11] and the Long-term Intervention with Pravastatin in Ischemic Disease (LIPID) study [12] showed that statin therapy for secondary prevention was comparably effective in older patients and their younger counterparts. Subanalyses of these studies designed to compare the effects of statin therapy in older and younger patients indicated that similar or greater benefit in mortality, major CV, events and stroke was obtained in patients who were 65 years of age or older at the time of enrolment [13–15]. Given the increased likelihood of CV events in older patients, a similar relative risk reduction with statin therapy confers a greater absolute benefit. In the CARE study, for instance, coronary death was reduced from 10.3% to 5.8% in patients over 65 years of age, whereas in younger patients no such absolute risk reduction was observed [13]. Likewise, in patients aged 65 to 75 years, stroke was reduced from 7.3% to 4.5% with pravastatin treatment, but in younger patients only a 0.4% absolute risk reduction was observed. Data from the Cardiovascular Health Study (CHS), an observational study which included largely a primary prevention population, showed a 56% reduction in CV events and a 44% reduction in all-cause mortality in patients 65 years or older who received statin therapy compared with those who did not receive statins [16]. Subsequent studies such as the Heart Protection Study (HPS) [17] and the lipid-lowering arm of the Anglo-Scandinavian Cardiac Outcomes Trial (ASCOT-LLA) [18] have shown similar results. Studies designed specifically to address older patients, such as the Prospective Study of Pravastatin in the Elderly at Risk (PROSPER) [19] also showed a significant reduction in the rate of CV events. The benefit of statin therapy for secondary prevention in older patients has recently been confirmed by a metaanalysis of nine clinical trials [20], which concluded that the reduction in mortality associated with statin therapy in older patients was much greater than previously estimated. A metaanalysis of 10 randomized clinical trials that enrolled 70 388 people with CV risk factors but without established CV disease showed comparable benefit in those younger and older than age 65: improved survival and reduction in the risk of major CV events [21]. Finally, the benefits of intensive statin regimens to achieve substantial LDL-C reductions are clearly maintained in patients aged over 65 years [22, 23]. Analysis of Kaplan-Meier plots to assess the effect of treatment on CV events over time suggests that in many cases benefit may be obtained relatively soon after initiation of statin therapy [22–24]; given the higher incidence of events in an older population, benefits are likely to be even more apparent than in younger individuals.

The data on the benefit of statin therapy in older patients are compelling and support the assertion in the National Cholesterol Education Program Adult Treatment Panel III (NCEP ATP III) guidelines that age should not be a barrier to such treatment [3, 4]. Nevertheless, older patients commonly remain untreated or undertreated [25, 26]. Following the update to NCEP ATP III in 2004, analysis showed that among very-high-risk older individuals, 85% of patients newly initiated to statin therapy and 78% of ongoing users received minimal-guideline therapy [26]. Furthermore, <50% of very-high-risk older patients attained the optional goal of <70 mg/dL.

A number of factors may contribute to this failure to provide appropriate statin therapy. First, despite evidence of clear benefit in older patients, the proportion of such patients enrolled in pivotal statin trials has often been relatively low, particularly those above 70 years of age (Table 1). The lower proportion of older patients in these studies may lead to an inaccurate perception regarding the strength of the data available on statin use in older age individuals, with uncertainty arising among physicians and payers, as well as older patients and their families. Older patients may also be expected to have diminished liver and kidney function, small stature and general frailty. As a result of such generalization, high-dose statins are commonly perceived as more likely to cause adverse events in older patients, despite a lack of evidence to support such an assertion, and this may contribute to the undertreatment of hyperlipidemia in this patient group. In addition, older patients are often prescribed multiple medications and the potential for drug-drug interactions that could limit the use of certain statins may be of particular concern. All these factors may begin to explain why older patients are less likely to be prescribed statins and, even when prescribed, less likely to be titrated to doses sufficient to achieve LDL-C targets [25].

To address the problem of undertreatment in older patients, physicians must tailor treatment based on the overall CV risk of each individual, regardless of age. Appropriate management involves balancing the need to treat to guideline-based LDL-C targets with the general safety concerns that influence the treatment of older patients and an assessment of the general health and prognosis of each individual. In other words, age should not constitute a barrier to appropriate statin treatment for the reduction of CV risk. The age-associated increase in CV risk is reflected in the inclusion of age as a significant variable in determining the Framingham risk score, which rises from middle age onwards [3, 27]. As stated in the NCEP ATP III guidelines, older age reflects the cumulative exposure to atherogenic risk factors [3]. However, an individual's overall CV risk is influenced by numerous other factors [28, 29]. Consequently, arbitrary decisions to alter treatment approaches in patients above a given age—whether 65, 75, or even 85 years—are inappropriate. Rather, age can be considered as simply another risk factor to be taken into account along with other factors relating to CV risk, general health, safety, and prognosis considered concomitantly in identifying the most appropriate treatment when assessing strategies for the prevention and treatment of CV disease. 

In this paper, hypothetical case studies are used to illustrate the assessment of CV risk in an older patient and to incorporate the appropriate use of guidelines and CV outcomes trial evidence to assist the treating physician's decision-making process. We attempt to answer the frequently asked questions of clinicians who manage the CV health of older patients.

## 2. Clinical Vignettes

### 2.1. Patient 1—Acute Coronary Syndrome

This 76-year-old white female is 5′3′′ tall and weighs 142 lbs; her body mass index (BMI) is 25.2. She is a nonsmoker with no history of diabetes. She was hospitalized 2 weeks previously with a non-ST-elevation myocardial infarction (NSTEMI) and underwent a percutaneous coronary intervention (PCI) with bare-metal stent placement. During the hospitalization she received aspirin 325 mg/d, clopidogrel 75 mg/d, metoprolol 50 mg/d, lisinopril 20 mg/d, and atorvastatin 80 mg/d. There was no heart failure or serious arrhythmia. At a cardiology follow-up appointment after discharge, it was found that the patient was receiving aspirin 81 mg/d, clopidogrel 75 mg/d, metoprolol 50 mg/d, lisinopril 20 mg/d, and had been changed by her primary care physician to simvastatin 20 mg/d. Laboratory studies indicated the following: total cholesterol (TC) = 180 mg/dL, LDL-C = 97 mg/dL, high-density lipoprotein cholesterol (HDL-C) = 39 mg/dL, triglycerides (TGs) = 154 mg/dL, and creatinine = 1.2 mg/dL. 

This patient with a recent acute coronary syndrome (ACS) is at very high risk of recurrent CV events. Both NCEP ATP III [4] and American College of Cardiology/American Heart Association (ACC/AHA) unstable angina/NSTEMI guidelines [5] support the optional LDL-C target of <70 mg/dL. However, the patient's current statin regimen (simvastatin 20 mg/d), initiated by her primary care physician following discharge, has not achieved this goal, and titration to a higher dose likely would be insufficient to achieve the LDL-C target or offer clear benefit in terms of CV outcomes. It is unclear why the hospital regimen of atorvastatin 80 mg/d was not continued. Based on the percentage reduction in LDL-C achieved with the current regimen (Table 2), the patient's unmedicated LDL-C would be expected to be approximately 156 mg/dL. Consequently, to achieve a target of <70 mg/dL a 56% reduction is required. As shown in Table 2, this likely would not be achieved with the highest dose of simvastatin (80 mg/d). Statin dose response tends to obey the rule of sixes, such that the greatest lipid-lowering response is obtained at the starting dose, while incremental doubling of the statin dose leads to an approximately 6% additive reduction in LDL-C. Titration to even the highest dose of simvastatin would not also be expected to provide additional CV benefit [30] and may be associated with a higher risk of myopathy [31]. This highlights the importance of appropriate choice of statin regimen, considering the predicted potency of statin dose and ensuring that an appropriate dose to achieve the patient's LDL-C target is employed from the outset. An evidence-based approach to treatment of this patient supports the use of intensive statin therapy. 

A statistically significant benefit of atorvastatin 80 mg/d to prevent major CV events in patients with ACS has been shown in both the Pravastatin or Atorvastatin Evaluation and Infection Therapy—Thrombolysis in Myocardial Infarction 22 (PROVE IT—TIMI 22) [33], and Myocardial Ischemia Reduction with Aggressive Cholesterol Lowering (MIRACL) [34] studies when compared with standard therapy (pravastatin 40 mg/d) or placebo, respectively. Analysis of patients at least 70 years of age in PROVE IT-TIMI 22 [35] and comparison of patients aged <65 or ≥65 years in MIRACL [36] showed that high-dose atorvastatin was as safe and effective in older as in younger patients. Also, a subanalysis of the Treating to New Targets (TNT) study showed that treatment with atorvastatin 80 mg continued to reduce the risk of any recurrent CV event over time compared with atorvastatin 10 mg in patients 65 years of age or older [37], suggesting that continuation of high-dose statin therapy would have sustained benefit in this patient. The cardiologist therefore determined that atorvastatin 80 mg/d should be reinstated and treatment continued with aspirin 81 mg/d, clopidogrel 75 mg/d, metoprolol 50 mg/d and lisinopril 20 mg/d.

In subsequent years, this patient would require ongoing followup for chronic CHD. The LDL-C targets specified in the NCEP ATP III guidelines for such patients remain similar (<100 mg/dL with an optional target of <70 mg/dL). Although a more moderate statin dose could be sufficient to achieve a goal of <100 mg/dL, data on clinical outcomes suggest that intensive lipid-lowering to achieve the optional target will have greater benefit without increasing the likelihood of adverse events. The TNT study showed a relative risk reduction of 19% for major CV events with atorvastatin 80 mg/d versus atorvastatin 10 mg/d in stable CHD patients 65 years of age or older, with no notable difference in withdrawal from therapy due to adverse events. A subanalysis of patients aged 65 to 78 years in the Aggressive Lipid-Lowering Initiation Abates New Cardiac Events (ALLIANCE) study also showed a significant benefit of titration to more intensive statin therapy [23]. This study compared the patient's usual care (continuation of baseline therapy with changes and laboratory studies as directed by their treating physicians) with titration to a mean atorvastatin dose of 36.5 ± 27.4 mg/d (59.9% were titrated to at least 40 mg/d and 38.7% to 80 mg/d), resulting in a 27% reduction in the relative risk of major CV events. Similarly, in the Study Assessing Goals in the Elderly (SAGE) [24], intensive atorvastatin therapy in patients aged 65 to 85 years reduced major CV events and all-cause mortality to a greater extent than moderate statin therapy with pravastatin. Taken together these findings support continuation of intensive statin therapy to achieve the optional LDL-C target of <70 mg/dL.

### 2.2. Patient 2—High-Risk Older Patient with Comorbidities

This 73-year-old African American male is 5′10′′ tall and weighs 216 lbs; his BMI is 31.0. He has a history of CHD (coronary artery bypass graft [CABG] 5 years previously) and mild-to-moderate chronic kidney disease (CKD), with an estimated glomerular filtration rate (eGFR) of 52 mL/min. He was diagnosed with type 2 diabetes at age 65 years and has metabolic syndrome according to the International Diabetes Federation definition [38], further increasing his CV risk. He discontinued cigarette smoking 5 years ago and has a family history of CV disease. There is no current angina or congestive heart failure. He has a blood pressure (BP) of 133/77 mmHg (medicated), TC = 178 mg/dL, LDL-C = 117 mg/dL, HDL-C = 44 mg/dL, TGs = 180 mg/dL, HbA1c = 7.3%, fasting glucose = 150 mg/dL, and serum creatinine = 1.4 mg/dL.

The patient had reasonable exercise tolerance prior to an episode of pneumonia, for which he was admitted to the emergency department, treated with azithromycin and rocephin, and discharged with a prescription for a quinolone. Prior to admission he was receiving atorvastatin 10 mg/d, but the physician treating his pneumonia discontinued statin treatment for the duration of the antibiotic therapy. The primary care physician saw the patient for a follow-up appointment after discharge and completion of the antibiotic course. Current medications are aspirin 81 mg/d, metformin 1000 mg/d, ramipril 10 mg/d, and carvedilol 25 mg twice daily. 

Although it is not unreasonable to discontinue statin therapy during treatment with a macrolide antibiotic, this should be a temporary discontinuation (drug holiday) and statins should be reinstated following completion of antibiotic therapy. In this high-risk patient with concomitant risk factors, the statin regimen prior to the episode of pneumonia was inadequate to reach his target LDL-C levels and should be reassessed. The updated NCEP ATP III guidelines indicate that such a patient should be treated to a LDL-C goal of <100 mg/dL with an optional goal of <70 mg/dL [4]. A higher dose of a potent statin may therefore be required. Strong evidence supporting intensive statin therapy in such a patient comes primarily from subanalyses of the TNT trial. Compared with patients receiving atorvastatin 10 mg, intensive therapy with atorvastatin 80 mg yielded significant benefits in TNT subjects who had either diabetes [39], CKD [40], or both [41]. These analyses also found no differences in the rate of adverse events compared with the total group of patients in TNT. The results of the TNT subanalysis in older patients [22] provide further support for the use of atorvastatin 80 mg in this patient. High-dose statin therapy may also benefit this patient by improving kidney function. Among patients from the TNT study with diabetes and CKD, those receiving atorvastatin 80 mg showed significantly greater improvement in kidney function than those treated with atorvastatin 10 mg (mean change in eGFR from baseline with atorvastatin 10 mg versus 80 mg: 0.5 versus 2.6 mL/min) [41]. Weight control should also be assessed and therapeutic lifestyle change prescribed through dietary improvement and regular exercise. Once the patient's LDL-C goal has been reached, if the TG levels are not <150 mg/d it may be appropriate to consider reducing his TG levels with addition of omega-3 fatty acids, niacin, or fenofibrate.

### 2.3. Patient 3—Primary Prevention

This 66-year-old male is 5′7′′ tall and weighs 179 lbs; his BMI is 28. He is a nonsmoker and is receiving treatment for hypertension. He has a BP of 139/85 mmHg, TC = 201 mg/dL, LDL-C = 119 mg/dL, HDL-C = 50.1 mg/dL, TGs = 150 mg/dL, and *C-*reactive protein (CRP) = 4.2 mg/L. The patient is very health conscious, has a good routine level of physical activity, and had his CRP level measured, at his own request, following media coverage of the Justification for the Use of Statins in Prevention: an Intervention Trial Evaluating Rosuvastatin (JUPITER) study [42]—the measurement was done only once at an off-site laboratory. Although his serum LDL-C level is relatively low, he has a Framingham risk score of 17%, and is therefore classified as at moderately high risk. He is currently treated with aspirin 81 mg/d, lisinopril 20 mg/d, and a thiazide diuretic 25 mg/d.

The findings of the ASCOT Blood Pressure Lowering Arm (ASCOT-BPLA) highlighted the importance of BP control in reducing CV risk [43]. In that study, many patients remained hypertensive despite receiving antihypertensive therapy prior to enrolment. Since this patient remains hypertensive with his current treatment regimen, the dose of lisinopril should be increased to 40 mg/d. In addition, the lipid-lowering arm of the ASCOT trial (ASCOT-LLA), in which hypertensive patients with a fasting LDL-C of ≤251 mg/dL were randomly assigned to treatment with atorvastatin 10 mg or placebo, showed a significant reduction in the risk of CV events with atorvastatin therapy [18]. Similar results were also obtained in a post hoc analysis of the ASCOT-LLA which showed that atorvastatin 10 mg significantly reduced the risk of CV events in patients 65 years of age or older [44]. Given the Framingham risk score of this patient, current guidelines and clinical trial evidence would support treating to an optional LDL-C goal of <100 mg/dL. As this corresponds to a 16% reduction in LDL-C, various lipid-lowering options are available (Table 2).

The results of the JUPITER trial further confirmed the benefit of lipid-lowering in moderately high-risk initially healthy patients and supported the need to treat such patients even more intensively. Compared with placebo, rosuvastatin 20 mg/d led to a 43% reduction in the risk of major CV events in patients with LDL-C <130 mg/dL and persistently elevated CRP (>2.0 mg/dL) [42]. There was no difference in the benefit for patients aged <65 or ≥65 years. Although the patient in this case study is comparable to subjects enrolled in the JUPITER study, elevated CRP was only documented in a single measurement. Since CRP is an acute phase reactant and its concentration increases rapidly in response to inflammation, the finding could easily be explained by a variety of factors, including simple infection. It would be necessary to assess CRP based on the average of two independent measurements, as was done in the JUPITER trial. Nevertheless, while the results of the JUPITER trial are provocative, they have not been incorporated into guidelines. The relevance of CRP levels is also currently under consideration and it remains to be seen whether this parameter will be included in future guidelines. This patient should therefore receive treatment based on current recommendations [4], to achieve the optional LDL-C goal of <100 mg/dL with an appropriate dose of statin. Importantly, the rule of sixes should be taken into account (the greatest lipid-lowering response is obtained at the initial statin dose, while incremental doubling of the dose leads to an approximately 6% additive reduction in LDL-C) and an appropriate starting dose used to achieve this target without a requirement for subsequent dose increase. However, if the LDL-C goal is not achieved after 6 to 8 weeks, the statin regimen should be changed accordingly.

### 2.4. Patient 4—Primary Prevention in an Ostensibly Heart-Healthy Older Patient

This 80-year-old Caucasian male continues to be an avid mountain climber and comes in for a medical evaluation prior to his next climb. During the examination he asks the physician if he should be taking a statin. He is 6′0′′ tall, weighs 172 lbs, and has a BMI of 23.3. He is a nonsmoker with no history of diabetes, and his only medication is aspirin 81 mg/d. However, his father died of a myocardial infarction at age 54, which has led him to maintain a healthy lifestyle. He has a BP of 120/75 mmHg, TC = 190 mg/dL, LDL-C = 140 mg/dL, HDL-C = 49 mg/dL, and TGs = 120 mg/dL.

Although this patient seems healthy and is very active, his Framingham risk is 17%, primarily due to his advanced age, indicating a moderately high risk of CV disease. Thus, even in an apparently healthy patient, age continues to be a very important risk factor. Although clinical trial data are scarce for primary prevention in subjects of this age, on the basis of risk, the NCEP ATP III update recommends an optional LDL-C goal of <100 mg/dL for this patient [4]. If the patient were hypertensive, this recommendation would be supported by the findings of the ASCOT-LLA [18]. In that study, patients had a mean baseline LDL-C just over 130 mg/dL; treatment with atorvastatin 10 mg/d versus placebo led to a significant reduction in CV risk, and this effect remained apparent in patients aged over 65 years [44]. Likewise, were the patient to have an elevated CRP, the results of the JUPITER [42] trial would have some bearing on his treatment. However, there have been no specific trials addressing patients of this type. Thus, in the absence of new recommendations, current guidelines should be followed and the patient should be treated to an LDL-C goal of <130 mg/dL or an optional goal of 100 mg/dL with an appropriate statin, titrated to goal. To reach the optional goal, it would be necessary to use a statin regimen that would allow at least a 29% reduction in LDL-C (Table 2). Consequently, a variety of options are available, including the use of generic statins.

## 3. Statin Therapy in Older Patients—Safety and Adherence

A number of statin clinical trials and subanalyses have shown that treatment-related adverse events are not more common in older patients compared with their younger counterparts. In the TNT study (*n* = 10 001), the proportion of patients aged ≥65 years (*n* = 3809) who experienced treatment-related adverse events was comparable to that observed in patients <65 years for both the 10 mg and 80 mg doses of atorvastatin [22]. In SAGE, which enrolled only older participants (range: 65–85 years), a similar proportion of patients experienced adverse events in the atorvastatin 80 mg and pravastatin 40 mg treatment arms [24]. As in other studies of high-dose statin regimens [45], the frequency of elevated liver enzymes remained low overall but elevated compared to less-intensive regimens (4.3% of patients treated with atorvastatin 80 mg versus 0.2% of patients treated with pravastatin 40 mg). Liver function should be monitored in all patients treated with high-dose statins. However, treatment decisions should be made on an individual basis and concerns about hepatotoxicity should not be an automatic barrier to intensive statin therapy to achieve LDL-C targets in older patients. Similarly, myalgia and myopathy appear to be associated with statin therapy, but do not seem to be increased in older patients compared with their younger counterparts [22, 24, 46].

The largest analysis of statin safety in older patients reported to date included data pooled from 50 clinical trials of atorvastatin therapy [47]. In that analysis, 5437 patients 65 years of age or older were found to have a low rate of serious adverse events (≤1%) and there was little difference in the proportion of patients who withdrew due to treatment-related adverse events associated with placebo or atorvastatin treatment (2.1% versus 1.7%). 

Elevated alanine transaminase or aspartate transaminase levels were more common in patients treated with atorvastatin 80 mg compared with placebo (3.2% versus 0.9%), but specific musculoskeletal or liver abnormalities remained rare (≤3%). A similar study involving a pooled analysis of 3145 patients aged ≥75 years who received placebo or atorvastatin 10–80 mg in 45 completed randomized trials demonstrated that the rate of adverse events did not increase with higher doses of the drug and was similar in atorvastatin-treated patients and those who received placebo [48]. Thus, currently available evidence suggests that the safety of statin therapy remains similar in older and younger patients, even with intensive lipid lowering.

Although age may not in itself represent a barrier to intensive statin therapy, a large proportion of older patients are receiving multiple medications for other conditions and care should be taken to avoid drug-drug interactions. As an example, older patients receiving concomitant amiodarone may be at particularly high risk of myopathy when treated with statins that are metabolized by CYP3A4 [49]. In such patients, it may be preferable to use pravastatin or rosuvastatin, as they are not metabolized by CYP3A4. Any drugs that bind enzymes involved in statin metabolism may be liable to alter statin levels and increase the risk of toxicity. In the case of the CYP3A4-metabolized statins, drugs include verapamil and diltiazem, which may be used with care in combination with low doses of those statins, or systemic azole antibiotics such as itraconazole or ritonavir, which are associated with a much higher risk of statin toxicity and should not be used in combination with statins [50, 51]. The possibility of drug interactions with warfarin should also be taken into account [52], and where necessary, alternatives such as atorvastatin considered [53]. In patients with mixed dyslipidemia, use of fenofibrate is preferable to gemfibrozil, since fenofibrate is not associated with significant inhibition of statin metabolism [54]. As in all patients, other possible interactions with foodstuffs or other products must be taken into consideration. Grapefruit juice is suggested to be a risk for statin toxicity through its inhibition of CYP3A4 [55], but is likely to be clinically relevant only when consumed in large quantities (>1.2 L/d). St. John's Wort has been shown to reduce plasma concentrations of simvastatin [56].

Adherence to statin therapy is relatively poor among all patients treated, but this phenomenon appears to be especially true in primary prevention patients [57] and older patients [57–59]. For example, a large cohort study of patients aged ≥65 years found that adherence declined according to risk—only 40.1% in ACS patients, 36.1% in patients with chronic CHD, and 25.4% in those treated for primary prevention [59]. Clearly, such low adherence results in limited benefit, such that it is important for primary care physicians to monitor and encourage treatment adherence carefully while ensuring that patients are treated to guideline-based goals. Progress towards LDL-C goals has a positive impact on adherence to statin therapy [60–62]. However, by exhibiting a greater tendency to arbitrarily withdraw intensive statin therapy or failing to titrate to target LDL-C levels in older patients, physicians may contribute to undertreatment. Adherence to both simvastatin 20−40 mg/d and atorvastatin 80 mg/d was generally very high (>80%) in patients ≥65 years of age enrolled in the open-label Incremental DEcrease through Aggressive Lipid Lowering (IDEAL) study [46]. However, despite no difference in the rate of adverse events, treatment withdrawal due to adverse events was significantly higher in patients randomized to atorvastatin therapy. Since most patients had received simvastatin prior to randomization, the authors of that study interpreted the increased tendency to withdraw a more potent statin dose as a possible reflection of greater reluctance of physicians to continue high-dose statin therapy in older patients. If treatment options are to be used effectively to reduce CV risk in an older patient population, physicians must ensure that, while carefully monitoring the progress of the individual patient, they follow appropriate treatment guidelines and base their clinical decisions on available evidence.

## 4. Conclusions

Age alone places older patients at increased risk of CV disease, independent of the presence or absence of other CV risk factors. Yet older individuals remain pervasively undertreated with lipid-lowering drugs. Even when prescribed lipid-lowering therapy, recommended LDL-C targets are rarely achieved. A large body of evidence supports the effectiveness of intensive statin therapy in older patients. However, concerns that safety may be a particular issue in an older population appear largely unfounded, so long as known issues (e.g., drug-drug interactions) are taken into consideration. If current lipid-lowering guidelines are followed and older patients are treated to appropriate LDL-C goals, the likelihood of CV events will be significantly reduced in this high-risk population.

## Figures and Tables

**Table 1:** Proportions of older patients enrolled in pivotal statin trials.

Study	Characteristics	Age at enrolment	
		≥65 years	≥70 years
Scandinavian Simvastatin Survival Study (4S) [10]	Simavastatin versus placebo Patients aged 35–70 years *n* = 4444	23%	—

Cholesterol and Recurring Events (CARE) Trial. Lewis et al. [13]	Pravastatin versus placebo Patients aged 21–75 years *n* = 4059	31%	12%

Heart Protection Study (HPS) [17]	Simavastatin versus placebo Patients aged 40–80 years *n* = 20 536	52%	28%

Treating to New Targets (TNT). Wenger et al. [22]	Atorvastatin 80 mg versus 10 mg Patients aged 35–75 years *n* = 10 001	38%	18%

**Table 2:** Percentage reductions in low-density lipoprotein cholesterol with different statin doses (Adapted from Alexander et al. [32]).

Statin	LDL-C reduction, %
Pravastatin	
10	22
20	32
40	34
80	37

Lovastatin	
10	21
20	27
40	31
80	42

Simvastatin	
5	26
10	30
20	38
40	41
80	47

Atorvastatin	
10	39
20	43
40	50
80	60

Ezetimibe/simvastatin	
10/10	45
10/20	52
10/40	55
10/80	60

Rosuvastatin	
5	45
10	52
20	55
40	63
